# Differential Impact of Adherence to Pegylated Interferon and Ribavirin in the Treatment of Genotype 1 High Viral Titer Chronic Hepatitis C

**DOI:** 10.1155/2010/702748

**Published:** 2010-08-23

**Authors:** Makoto Numata, Tatehiro Kagawa, Sei-ichiro Kojima, Shunji Hirose, Naruhiko Nagata, Koichi Shiraishi, Norihito Watanabe, Hirokazu Shiozawa, Yasuhiro Nishizaki, Shigeyuki Motegi, Shinji Takashimizu, Jun-ichiro Kamochi, Mitsuru Wasada, Takashi Ohno, Yoshihiro Tei, Atsushi Nakano, Takuji Yamada, Kazuhiro Atsukawa, Tetsu Watanabe, Tetsuya Mine

**Affiliations:** ^1^Department of Gastroenterology, Tokai University School of Medicine, Isehara 259-1193, Japan; ^2^Department of Gastroenterology, Tokai University Hachioji Hospital, Tokyo 192-0032, Japan; ^3^Department of Gastroenterology, Tokai University Tokyo Hospital, Tokyo 151-0053, Japan; ^4^Department of Gastroenterology, Tokai University Oiso Hospital, Oiso 259-0198, Japan; ^5^Ikegami General Hospital, Tokyo 146-8531, Japan; ^6^Isehara Kyodo Hospital, Isehara 259-1132, Japan; ^7^Japan Medical Alliance Ebina General Hospital, Ebina 243-0433, Japan; ^8^Tomei-Atsugi Hospital, Atsugi 243-8571, Japan; ^9^Hiratsuka City Hospital, Hiratsuka 254-0065, Japan; ^10^Department of Community Health, Tokai University School of Medicine, Isehara 259-1193, Japan

## Abstract

To clarify the impact of adherence, we treated 122 genotype 1 high viral titer chronic hepatitis C patients with pegylated interferon (peg-IFN) and ribavirin for 48 weeks at nine referral hospitals, and evaluated the prognostic factors with a focus on the adherence to the treatment. This study included 68 (55.7%) treatment-naïve patients and 54 (44.3%) patients who did not respond to the previous treatment. Multivariate analysis revealed adherence to peg-IFN and ribavirin as the only significant predictor. Sustained virological response (SVR) rate was 72.2%, 19.0%, and 27.3% in patients given ≥80%, 60%–80%, and <60% dose peg-IFN, respectively, and was 68.6%, 41.2%, and 5.3% in those given ≥80%, 60%–80%, and <60% dose ribavirin, respectively. SVR rate sharply fell when exposure to peg-IFN was below 80% whereas it decreased in a stepwise manner as for ribavirin. Therefore, ≥80% of peg-IFN and as much as possible dose of ribavirin are desired to achieve SVR in the treatment of genotype 1 high viral titer chronic hepatitis C.

## 1. Introduction

 Although the combination of pegylated interferon (peg-IFN) and ribavirin (RBV) is the standard-of-care therapy for chronic hepatitis C, the sustained virological response (SVR) rate is still 40%–50% [1–3] for patients who are infected with genotype 1 and have high viral load in their sera. Adherence to the therapy is an important factor associated with a favorable outcome. McHutchison et al. reported that the patients who received ≥80% of the scheduled doses of peg-IFN and RBV for ≥80% of the planned duration of therapy had an SVR rate of 51% compared with 34% in less adherent patients [4]. In contrast, a study on patients with advanced fibrosis revealed that reducing RBV dose did not affect SVR rate as long as peg-IFN dose was maintained [5]. Reddy et al. also reported that SVR rate was affected adversely by RBV dose reduction when cumulative exposure was less than 60%, and that RBV dose reduction raised the relapse rate [6]. The significant impact of adherence to both peg-IFN and RBV on SVR is well understood, however, there may be difference between these two drugs in the way they effect the response.

 Until now, many host factors including younger age (40 years or less) [2], female gender [7], lighter body weight [1, 2], the absence of insulin resistance [8], elevated ALT levels [2], less advanced liver histology [2, 7], and non-African American race [7, 9] are reported to be associated with favorable response. Recently the association of genetic variation of *IL28B* with response has been reported [10–12]. 

 Japanese elderly women were reported to be resistant to this therapy [13, 14]. Japanese patients are approximately 10 years older than those in other countries and our reports would provide useful information when considering therapy for elderly patients in other countries. The lower SVR rate in elderly women might be attributable to lower adherence to peg-IFN or RBV. However, few studies analyzed relationship between SVR rate and the adherence in elderly patients.

 In this study, we treated genotype 1 high viral titer chronic hepatitis C patients with peg-IFN and RBV combination therapy, and evaluated the prognostic factors with a focus on the adherence to the treatment.

## 2. Materials and Methods

### 2.1. Patients

 This study was performed at nine referral hospitals. Patients with hepatitis C virus (HCV) genotype 1 and high viral load (≥100,000 IU/mL) who received peg-IFN alfa-2b (Pegintron, Schering-Plough Corporation, Kenilworth, NJ) and RBV (Rebetol, Schering-Plough Corporation) combination therapy for 48 weeks from January 2004 to December 2006 were consecutively enrolled into the study. Exclusion criteria were as follows: (1) patients with leukopenia (<3,000/*μ*L), neutropenia (<1,500/*μ*L), thrombocytopenia (<90,000/*μ*L), or anemia (hemoglobin concentration <12 g/dL), (2) patients with creatinine clearance <50 mL/min, and (3) existence of cirrhosis, autoimmune diseases, uncontrolled mental disorders, uncontrolled malignancy, or severe heart or lung diseases. Written informed consent was obtained from all patients.

### 2.2. Treatment

 The patients were given peg-IFN alfa-2b at a dosage of 1.5 mg/kg every week subcutaneously for 48 weeks. Daily RBV was administered orally for 48 weeks according to the labeling approved by the Japanese Ministry of Health, Labour and Welfare; 600 mg for patients ≤60 kg, 800 mg for patients weighing 60 to 80 kg, and 1000 mg for patients >80 kg. The use of hematopoietic growth factors such as G-CSF and erythropoietin was not permitted in this study. Blood samples were collected every four weeks and parameters including complete blood cell counts, biochemistries, and the amount of HCV-RNA were determined. HCV serotype was tested with a serological genotyping assay kit (Immunocheck F-HCV Grouping; International Reagents Co., Tokyo, Japan) [15]. If HCV serotype was not definitive, HCV genotyping was performed (HCV Core Genotype; SRL, Tokyo, Japan). The response to the treatment was evaluated by an intention-to-treat analysis.

### 2.3. Statistical Analysis

 The factors associated with SVR were analyzed by logistic regression using SPSS version 16 (SPSS Japan, Tokyo, Japan). Univariate or multivariate logistic regression analyses were performed to establish the factors contributing to SVR. All reported *P*-values are 2-sided, with *P* < .05 considered statistically significant. The difference in the rates of relapse or SVR was evaluated by chi-square test.

## 3. Results

### 3.1. SVR

 A total of 122 patients were enrolled into the study. Forty-five patients (36.9%) were female and mean ± standard deviation (S.D.) of age was 54.0 ± 10.6 (min 19–max 70) years. Sixty-eight patients (55.7%) were naïve patients. The mean ± S.D. of weight and body mass index (BMI) was 63.5 ± 11.2 kg and 23.7 ± 3.3, respectively. High (100,000–800,000 IU/mL) and very high (≥800,000 IU/mL) HCV-RNA levels were observed in 36 (29.5%) and 86 (70.5%) patients, respectively. This study included 68 (55.7%) treatment-naïve patients and 54 (44.3%) patients who did not respond to the previous treatment. The previous treatment included a 24-week course of IFN alfa-2b and RBV combination therapy for 36 patients and a 24-week course of IFN alfa-2b or natural IFN alfa (human lymphoblastoid IFN) monotherapy for 18 patients. Forty-seven patients relapsed after the discontinuation of treatment, and the other 7 patients were nonresponders, in whom serum HCV-RNA were positive throughout the treatment. The SVR rate was 60.3%, 51.1%, and 28.6% in naïve patients, those with relapse, and nonresponders, respectively. In this study, the SVR rate was not significantly different between naïve patients and those treated previously. Liver biopsy was performed before treatment in 87 (71.3%) patients; 75 (86.2%) and 12 (13.8%) patients revealed METAVIR fibrosis score of 0–2 and 3-4, respectively. The SVR rate was not significantly different between these two groups; 57.3% in patients with F0–2 and 41.7% in those with F3-4. Finally 67 patients (54.9%) achieved SVR in the entire cohort.

### 3.2. Factors Associated with SVR (Table 1)

 Analyzed factors included gender, age, body weight, BMI, viral load, history of IFN treatment, and adherence to the treatment. Younger age, heavier weight, lower viral load, peg-IFN adherence, and RBV adherence were significant factors associated with SVR by univariate analysis. Multivariate analysis revealed adherence to peg-IFN and adherence to RBV as a significant predictor. We performed the same analysis after stratifying treatment-naïve and previously treated patients, and found adherence to peg-IFN and RBV as only factors significantly associated with SVR (data not shown) as shown in the entire cohort. 

**Figure 1:**
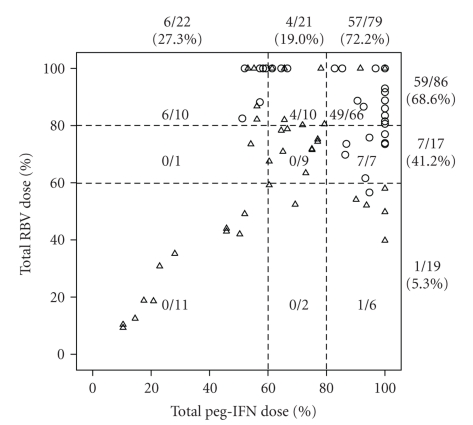
Scatter plot of patients with or without SVR according to administered total doses of peg-IFN and RBV. One hundred % represents a full scheduled dose. A circle and a triangle indicate a patient with SVR and one without SVR, respectively. A number represents number of patients with SVR/total number (SVR rate).

**Figure 2:**
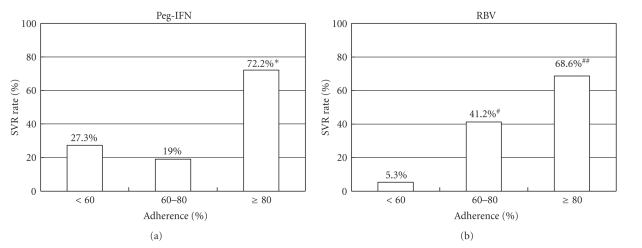
SVR rates classified by adherence to peg-IFN and RBV. *The SVR rate in patients with ≥80% dose of peg-IFN was significantly higher than that in those with <60% and 60%–80% (*P* < .001 for both). ^#^The SVR rate in patients with 60%–80% dose of RBV was significantly higher than that in those with <60% (*P* < .05). ^##^The SVR rate in patients with ≥80% dose of RBV was significantly higher than that in those with <60% (*P* < .001) and 60%–80% (*P* < .05).

 Patients given ≥80% dose of scheduled peg-IFN were more likely to achieve SVR by 7.7-fold (95% CI; 1.926–30.798, *P* = .004) than those given 60%–80% dose. The SVR rate in patients given 60%–80% dose peg-IFN was similar with those given <60% dose. Patients given ≥80% dose and those given 60%–80% dose of scheduled RBV were more likely to obtain SVR than those given <60% by 27.4-fold (95% CI; 3.130–240.151, *P* = .003) and by 15.7-fold (95% CI; 1.289–190.653, *P* = .031), respectively. The outcome of each case was shown in a scatter plot (Figure 1). The SVR was 1/19 (5.3%), 7/17 (41.2%), and 59/86 (68.6%) in patients given <60%, 60%–80%, and ≥80% of total RBV dose, respectively (Figure 2). Therefore, the more RBV was administered, the higher was the SVR rate. On the other hand, SVR was achieved in 6/22 (27.3%), 4/21 (19.0%), and 57/79 (72.2%) patients given <60%, 60%–80%, and ≥80% of total peg-IFN dose, respectively. Peg-IFN dose of 80% or more was important to obtain SVR. Notably none of the patients who received <80% dose for both drugs resulted in SVR (Figure 1). The relationship between SVR and adherence was analyzed separately in the treatment-naïve group and the previously treated group. In the treatment-naïve group the SVR rate was 74.5%, 20.0%, and 36.4% in patients given ≥80%, 60%–80%, and <60% dose peg-IFN, respectively, and was 74.5%, 54.5%, and 0% in those given ≥80%, 60%–80%, and <60% dose RBV, respectively. In the previously treated group, SVR rate was 68.8%, 18.2%, and 18.2% in patients given ≥80%, 60%–80%, and <60% dose peg-IFN, respectively, and was 61.3%, 33.3%, and 11.1% in those given ≥80%, 60–80%, and <60% dose RBV, respectively. These trends were similar with the results obtained from the entire cohort.

 There was a trend that younger patients received greater peg-IFN dose; 72/106 (67.9%) patients younger than 65 years and 7/16 (43.8%) patients aged 65 or older received ≥80% of total peg-IFN dose (*P* = .059). 

 Sixty-six patients (54.1%) received ≥80% dose for both drugs. Of these 49 (74.2%) patients resulted in SVR. When analysis was performed in these patients, no significant factors associated with SVR were chosen.

### 3.3. Rapid Virological Response (RVR), Early Virological Response (EVR), and Relapse

**Figure 3:**
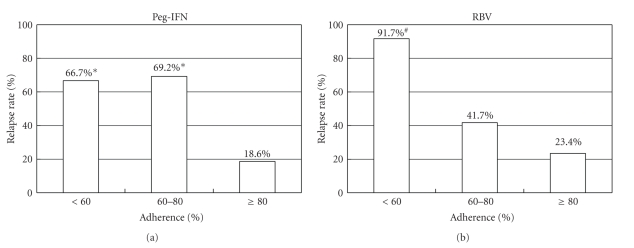
Relapse rates classified by adherence to peg-IFN and RBV. *The relapse rate in patients with <60% and 60%–80% dose of peg-IFN was significantly higher than that in those with ≥80% (*P* < .001 for both). ^#^The relapse rate in patients with <60% dose of RBV was significantly higher than that in those with 60%–80% (*P* < .05) and ≥80% (*P* < .001).

 The population of patients whose serum HCV-RNA first disappeared at week 4 (RVR), week 8, week 12 (EVR), week 24, and week 48 was 10 (8.2%), 39 (32.0%), 28 (23.0%), 20 (16.4%), and 4 (3.3%) patients, respectively. Twenty-one (17.2%) patients were positive for HCV-RNA throughout the treatment period (null response). The SVR rate of these patients who became negative for HCV-RNA at week 4 (RVR), week 8, week 12 (EVR), week 24, and week 48 was 10/10 (100%), 35/39 (89.7%), 17/28 (60.7%), 5/20 (25%), and 0/4 (0%), respectively. In 101 patients negative for HCV-RNA at the end of treatment, 34 (33.7%) patients relapsed. Relapse rate was significantly lower in patients who received ≥80% dose of peg-IFN than that in those who received 60%–80% or <60% dose (18.6% in patients with ≥80% dose versus 69.2% in those with 60%–80% dose (*P* < .001) and 66.7% in those with <60% dose (*P* < .001), Figure 3). The relapse rate increased in a stepwise fashion according to the adherence to RBV (91.7% in patients with <60% dose versus 41.7% in those with 60%–80% dose (*P* < .05), and versus 23.4% in those with ≥80% dose (*P* < .001)). These results were inversely associated with SVR rates.

### 3.4. Adverse Effect

 Seventeen (13.9%) patients discontinued treatment. The reasons of premature discontinuation were general fatigue and/or appetite loss (11 patients), fundal hemorrhage (1 patient), deterioration of diabetes mellitus (1 patient), and depression (1 patient). Three patients discontinued treatment because of positive HCV-RNA at week 24. Thirty-nine (32.0%) and 33 (27.0%) patients required dose reduction of peg-IFN and RBV, respectively. Major reasons of dose reduction were neutropenia or thrombocytopenia for peg-IFN and anemia for RBV. Common adverse effects included general fatigue, appetite loss, weight loss, and pruritus. In 12 patients with advanced liver disease (METAVIR fibrosis score of 3-4), 6 (50%) and 4 (33.3%) patients required dose reduction of peg-IFN and RBV, respectively. In 75 patients with milder liver disease (METAVIR fibrosis score of 0–2), 22 (29.3%) and 20 (26.7%) patients required dose reduction of peg-IFN and RBV, respectively. There was no significant difference between these two groups in the proportion of patients who required dose reduction.

## 4. Discussion

 The mean age of our study population was 54.0 years, which was approximately 10 years older than patients of major studies in Western countries [1–3]. Our cohort consisted of treatment-naïve patients (55.7%) and those who did not respond to the prior treatment (44.3%). SVR was achieved in 54.9% patients.

 In our study, adherence to peg-IFN and RBV was the only significant factor associated with SVR. Interestingly, the SVR rate stepwisely rose by the increase of administered dose of RBV. In contrast, 80% or more dose of peg-IFN was required to achieve SVR (Figure 2). This observation resulted from the likelihood of relapse (Figure 3); higher relapse rate was documented in a stepwise fashion in patients with smaller exposure to RBV, as previously suggested [16–18]. SVR rate was 74.2% when both drugs were administered ≥80%. Notably none of the patients who received <80% dose of both drugs attained SVR (Figure 1), confirming the validity of 80/80/80 rule together with ≥80% treatment duration.

 The difference between peg-IFN and RBV in the impact of adherence on SVR, especially within the <80% dose range, is still unclear. In our study, SVR rate sharply fell when exposure to peg-IFN was below 80% whereas it decreased in a stepwise manner as for RBV. Hiramatsu et al. recently reported that RBV dose reduction raised relapse rate in a dose-dependent manner [19], which is in agreement with our results.

 At least 80% dose of peg-IFN will be necessary to obtain favorable outcome. In contrast, RBV should be administered as much as possible within the planned dose. To accomplish this, RBV dose should be reduced by 200-mg decrements when anemia appears, and restored to the previous dose when anemia improves. Higher than standard dose RBV given together with standard dose peg-IFN may increase SVR rate [20], however, safety issues such as severe anemia are the major concern for this approach. Although the use of erythropoietin contributes to maintain RBV dose, the effect on SVR has not been shown [21, 22]. 

 Sezaki et al. reported that elderly women were resistant to peg-IFN and RBV combination therapy in Japan [13, 14]. In our study, younger age was a significant factor by univariate analysis, however, neither gender nor age was significantly associated with SVR by multivariate analysis. There was a trend towards lower adherence to peg-IFN in elderly patients. Therefore, older age itself is not a significant factor but is related to dose reduction or discontinuation, as reported by Iwasaki et al. [23]. 

 SVR rate was 74.2% when both drugs were administered ≥80%. Japanese patients are approximately 10 years older than those in other countries and anticipated to be vulnerable to adverse effects. Therefore, the adjuvant therapy that alleviates adverse effects should be developed. We recently demonstrated that maloxicam, a COX-2 inhibitor, decreased the rate of patients who required dose reduction by preventing the decrease of neutrophil counts [24]. 

 In this study, serotyping was used instead of genotyping because genotyping was not covered by the Japanese national health insurance. Serotype 1 includes genotype 1a and 1b. Because genotype 1a is rarely observed in Japan [25], most patients of this study are assumed infected with genotype 1b. Limitation of this study is a retrospective analysis with relatively small number of patients. Other major limitations are that our study consisted of a heterogeneous cohort (treatment-naïve and previously treated patients) and that liver histology was not available in approximately one third of the patients.

 In conclusion, 80% or more dose of peg-IFN and as much as possible dose of RBV are desired to achieve SVR in the treatment of genotype 1 high viral titer chronic hepatitis C.

## Figures and Tables

**Table 1:** 

Variables	SVR	*P*-value	Adjusted OR (95% C.I.)	*P*-value
Sex		.091		.501
Female	20/45 (44.4%)		1.00	
Male	47/77 (61.0%)		1.429 (0.506–4.032)	

Age (yr)		.019		.398
65<	5/16 (31.3%)		1.00	
51< ≤64	35/68 (51.5%)		2.655 (0.581–12.132)	
≤50	27/38 (71.1%)		2.695 (0.574–12.659)	

Weight (Kg)		.028		.116
<65	31/68 (45.6%)		1.000	
65≤	36/54 (66.7%)		3.053 (0.760–12.274)	

BMI		.716		.158
24≤	30/57 (52.6%)		1.000	
<24	37/65 (56.9%)		2.747 (0.674–11.236)	

Viral load (IU/mL)		.015		.174
800,000≤	41/86 (47.7%)		1.000	
100,000≤ <800,000	26/36 (72.2%)		2.137 (0.716–6.369)	

History of IFN treatment		.203		.581
yes	26/54 (48.1%)		1.000	
no	41/68 (60.3%)		1.316 (0.496–3.493)	

Peg-IFN adherence (%)		<.001		.008
<60	6/22 (27.3%)		2.637 (0.448–15.513)	.284
60≤ <80	4/21 (19.0%)		1.000	—
80≤	57/79 (72.2%)		7.702 (1.926–30.798)	.004

RBV adherence (%)		<.001		.010
<60	1/19 (5.3%)		1.000	—
60≤ <80	7/17 (41.2%)		15.679 (1.289–190.653)	.031
80≤	59/86 (68.6%)		27.416 (3.130–240.151)	.003
